# Evaluation of the Oxidative Status of Salami Packaged with an Active Whey Protein Film

**DOI:** 10.3390/foods8090387

**Published:** 2019-09-03

**Authors:** Mariana A. Andrade, Regiane Ribeiro-Santos, Manuela Guerra, Ana Sanches-Silva

**Affiliations:** 1 Department of Food and Nutrition, National Institute of Health Doutor Ricardo Jorge, I.P., Av. Padre Cruz, 1649-016 Lisbon, Portugal (M.A.A.) (R.R.-S.); 2 Department of Food Technology, Institute of Technology, Federal Rural University of Rio de Janeiro, Seropédica 23890-000, Brazil; 3 Estoril Higher Institute for Tourism and Hotel Studies, Av. Condes de Barcelona, 808, 2769-510 Estoril, Portugal; 4 National Institute for Agricultural and Veterinary Research (INIAV), I.P., Vairão, 4485-655 Vila do Conde, Portugal; 5 Center for Study in Animal Science (CECA), University of Oporto, 4050-313 Oporto, Portugal

**Keywords:** Aromatic plants, Food packaging, Lipid oxidation, Meat, Sensory analyses, Whey protein

## Abstract

Active packaging aims to prolong food’s shelf-life by directly interacting with the packaged food. This type of packaging is characterized by having the active agent incorporated into the package polymer, such as antioxidant additives, that will gradually migrate from the package polymer to the packed food and, consequently, delay food’s natural lipid oxidation. In this study, the efficiency of an active whey protein film incorporated with a rosemary extract on retarding the lipid oxidation of salami slices was evaluated. The lipid oxidation of the salami was measured by the thiobarbituric acid reactive substances (TBARS) assay and hexanal monitorization. Also, a sensory analysis on the salami packaged for 60 and 90 days was performed. The active film was able to delay the salami’s lipid oxidation for, at least, 30 days. The samples packaged with the active film revealed a bitter taste related to the rosemary extract and a bit sweet from the WP and the glycerol.

## 1. Introduction

In addition to contributing to the nutritional value of foods, lipids are essential for a healthy diet and offer foods characteristics such as texture, softness, aroma and taste that are important to consumers [1,2,3]. Foods with high lipid content are very susceptible to oxidation, especially when stored in the presence of light, oxygen, moisture and high temperatures [4].

Being one of the major causes of food deterioration, lipid oxidation can lead to considerable food industry economic losses. At molecular level, lipid oxidation may cause changes in the type and in the concentration of certain chemical compounds of foods, resulting in changes in the foods’ nutritional value, causing the loss of liposoluble vitamins, and organoleptic properties, leading to the formation of unpleasant tastes and/or aromas, reducing the food shelf life. In meat products, lipid oxidation represents a major problem, since it starts after slaughtering and it does not stop as long as there are fatty acids, continuing during post-mortem period, storage and processing [1,3]. The chemical compounds formed by the oxidation of lipids may represent a hazard to the human health since these compounds are linked to aging, carcinogenesis, atherosclerosis, mutagenesis, neurodegenerative diseases (such as Alzheimer’s disease and Parkinson’s disease) and inflammatory chronic diseases [1,2,4,5].

With the goal of delaying or inhibit the lipid oxidation, the food industry resorts to compounds with antioxidant activity, which can be synthetic or from natural origin and can be directly or indirectly applied to foods. The use of synthetic additives, like butylated hydroxytoluene (BHT) or butylated hydroxyanisole (BHA), in food preparations have been associated with the promotion of carcinogenesis, although they are stable and economic [6,7]. Thus, the interest in antioxidants from natural sources like aromatic plants is growing. Aromatic plants are constituted by active compounds, such as vitamins and phenolic compounds, which have important biological properties and are responsible for the plant’s defence against predators, radiation, drying periods, among others. The antioxidant power of aromatic plants is related to the presence of phenolic compounds. The use of these aromatic plants extracts in the food industry is being boosted from the consumer demand for more natural products [8,9,10]. The Food and Drug Administration have already recognized more than 150 extracts and essential oils from these plants as Generally Recognized as Safe (GRAS) [11]. The use of rosemary (*Rosmarinus officinalis* L.) extract as a food additive is authorized by the European Commission in the directives 2010/67/EU and 2010/69/EU [12,13], which can be directly applied to foods or incorporated into active food packaging. The principal phenolic compounds of the rosemary are rosmarinic acid, carnosic acid and carnosol, which is an oxidative hydroxylated derivative of carnosic acid [14,15].

The main function of the conventional food packaging is the protection of foods against external factors (temperature variations, radiation, light, microbials, among others), facilitate the transport and handling and inform consumers about food composition and nutritional value. Thus, the foods’ shelf-life is extended, and the microbiological safety is assured [16,17]. However, in addition to representing an environmental problem since most packages are made from polymers coming from non-renewable resources or non-biodegradable, they are just barriers that try to protect foods against the adverse effects of the food surrounding environment and attention shall be made to the migration of possible contaminants from packaging to food [16,18,19].

In this line of though, the active food packages emerged in order to protect foods against their natural degradation, extending their shelf-life by interaction with foods, maintain or improving their nutritional value and organoleptic properties [17,20,21]. These packages can also be environmentally friendly since they can be manufactured with polymers from renewable sources and/or biodegradable, such as whey protein [22].

Whey protein is a by-product of the dairy industry used in pastry and bakery products, infant formulas, sports drinks, ice-creams manufacturing, among others. The films made from whey protein are biodegradable and edible, therefore they are a green solution [23,24,25,26].

The main objective of this study is to evaluate the effectiveness against the lipid oxidation of a model food packaged with a whey protein film incorporated with rosemary extract. The evaluation of the effectiveness against lipid oxidation was evaluated through the TBARS assay and monitorization of the hexanal content. A sensory analysis was also performed to evaluate the influence of the rosemary extract in the aroma and taste of the model food.

## 2. Materials and Methods

### 2.1. Whey Protein Film 

For the rosemary extraction, the method described by Andrade et al. [27] was applied. Briefly, 5 g of dried powdered rosemary was mixed with 50 mL of ethanol. The solution was homogenized in a compact stirrer for 30 min at 350 rpm. Then, the solution was centrifuged, and the supernatant was removed to an evaporation flask. Ethanol was evaporated using a rotary evaporator at 35 °C, until dryness, and the extract was removed with the aid of a spatula.

Salami was the food chosen to evaluate the effectiveness of a whey protein film incorporated with a rosemary extract to inhibit lipid oxidation. The whey protein film is composed by 8% (*w*/*w*) of whey protein concentrate (WPC), 8% (*w*/*w*) of glycerol, 1% (*w*/*w*) of rosemary extract and 83% (*w*/*w*) of ultrapure water. Briefly, the WPC was mixed with ultrapure water and heated at 80 °C for 30 min. Then, the mixture was rapidly cooled to room temperature and glycerol and rosemary extract were added. The solution was homogenized and casted into an aluminium foil surface. The manufacture of the whey protein film, as well as the rosemary extract preparation, is based on previous work from our team [27].

### 2.2. Salami Slices

Salami was acquired already sliced in a commercial store in Lisbon, Portugal. The slices had, approximately, 20 g each. When acquired, they were stored in a polyethylene bag under vacuum at −20 °C, protected from the light. The nutritional composition is resumed in Table 1. According to the label, the salami was constituted by pork meat, pork loin, gelatine, milk powder, corn glucose syrup, soy protein, spices and salt. Also, the preservatives E-250 (Sodium nitrite—NaNO_2_) and E-252 (Potassium nitrate—KNO_3_), as well as the antioxidants E-316 (Sodium Erythorbate—C_6_H_7_NaO_6_) and the food coloring E-120 (Carmine acid—C_22_H_20_O_13_), were present.

**Figure 1:**
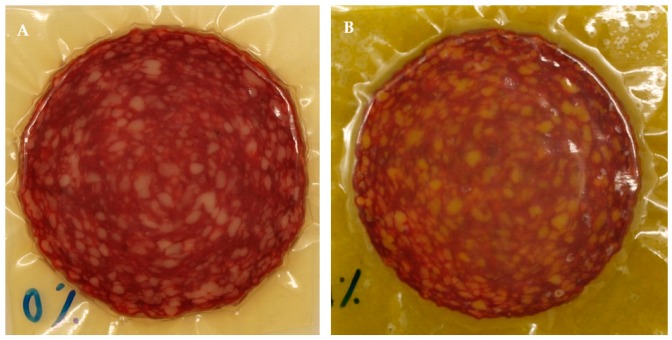
Salami slices packed with the control film (**A**) and the active film (**B**).

Both sides of each salami slice were placed in direct contact with the control film (without the rosemary extract) or with the active whey protein film (with 1% (*w*/*w*) of the rosemary extract) (Figure 1). Then, each slice of salami was placed inside polyethylene bags and these were packaged in vacuum conditions to allow a good contact between the film and the food. The salami slices were in contact with the active film for different storage times (0, 7, 15, 30, 60 and 90 days), protected from light and at 5 °C. Four slices of salami were packaged per treatment and per time period.

After the selected storage periods, the salami slices were separated from the packaging and homogenized separately with a Grindomix GM200 granulating mill (Haan, Germany) and stored separately at −80 °C under vacuum conditions in polyethylene bags, protected from the light, until analysis.

### 2.3. Lipid Oxidation Status of the Model Food

#### 2.3.1. Monitorization of Hexanal Content

The preparation of the samples was performed in accordance with the method developed by Wen, Morrissey, Walton, & Sheehy [29] and the hexanal identification and quantification was adapted from the method developed by Sanches-Silva et al. [30]. For the sample preparation, 1 g of the salami was homogenized with 5 mL of 1.7 mg/ mL of a 2,4-DNPH solution in a aqueous solution of sulfuric acid (30% (*w*/*w*)), at 8000 rpm for 2 min at room temperature, using an Ultra-Turrax IKA^®^ DI 25basic (Staufen, Germany). The solution was kept for 4 h at room temperature protected from the light. Then, 10 mL of *n*-hexane were added and the solution was centrifuged at 1914 g for 10 min at 15 °C. The supernatant was removed to an evaporator flask and the process was repeated twice. Next, *n*-hexane was evaporated at 35 °C and the residue re-dissolved in 10 mL of methanol. After filtration, the solution was analyzed in an UPLC^®^ ACQUITY™ (Waters, Milford, MA, EUA) equipped with a DAD detector. The pre-column was an ACQUITY™ UPLC^®^ BEH C18 (2.1 × 5.0 mm, 1.7 µm particle size) and the column was an ACQUITY™ UPLC^®^ BEH C18 (2.1 × 50 mm, 1.7 µm particle size).

Regarding the chromatographic method used to identify and quantify the hexanal, the mobile phases used were ultrapure water (Solvent A) and acetonitrile (Solvent B) in the proportion 25:75, in isocratic mode. Two mobile phases were filtered and degassed for 15 min. The column was maintained at 20 °C. The mobile phase flow was 0.5 mL/min and the injection volume was 10 μL. The hexanal was identified and quantified at 365 nm, at the retention time of 0.95 ± 0.1 min.

#### 2.3.2. TBARS Assay

The method used in this study was developed by Miller [31]. To 5 g of the homogeneized salami sample, 50 mL of 0.1 g/mL trichloroacetic acid dissolved in an aqueous solution of orthophosphoric acid (0.02 M) were added. The solution was homogenized with an Ultra-Turrax IKA^®^ DI 25basic (Staufen, Germany) for 1 min at 8000 rpm and filtered through a Whatman No 1. Then, to 5 mL of the filtered solution, 5 mL of a 2.9 mg/mL thiobarbituric acid (TBA) solution were added and the samples were submitted to 100 °C for 40 min in a heating block QBD2 from Grant Instruments (Cambridge, England). The control assay was composed of 5 mL of ultrapure water and 5 mL of the TBA solution. Then, the samples were rapidly cooled down to room temperature and their absorbance was measured in a spectrophotometer U-2000 from Hitachi at 530 nm, against the control assay.

### 2.4. Sensorial Analyses

In order to obtain a full understanding of the effect of the active film in the salami, a descriptive sensorial analysis was performed. To elect a panel of tasters, an online questionnaire was made available for consumers. The questionnaire included sociodemographic questions and participation requirements for the panel of tasters. Eighteen responses in the online questionnaire were obtained, but only 12 individuals were selected for the tastings. Four out of the 12 individuals were between 18 and 25 years old, 2 between 26 and 35 years old, 1 between 36 and 45 years old and 5 between 46 and 65 years old. Regarding the education level, 5 individuals owned a high school level, 4 individuals a bachelor’s degree, 2 individuals a master’s degree and 1 a PhD. Three sensorial sessions were performed. In the first session, the project was explained to the panel individuals and salami slices, acquired in the same day, were tasted and the evaluation descriptors were defined. The panel used a 1 to 5 descriptive scale, where 1 was used for the lowest intensity and 5 for the strongest intensity. In this session 15 descriptors were defined to analyze the samples in the following sessions. Regarding taste, 7 descriptors were defined: salty, smoked, strange, acid, bitter, spicy and paprika. Regarding the texture, 4 descriptors were defined: greasy, fibrous, juicy and softness. Finally, 4 more descriptors were defined: red color, typical aroma, typical odor and appealing aspect.

**Figure 2:**
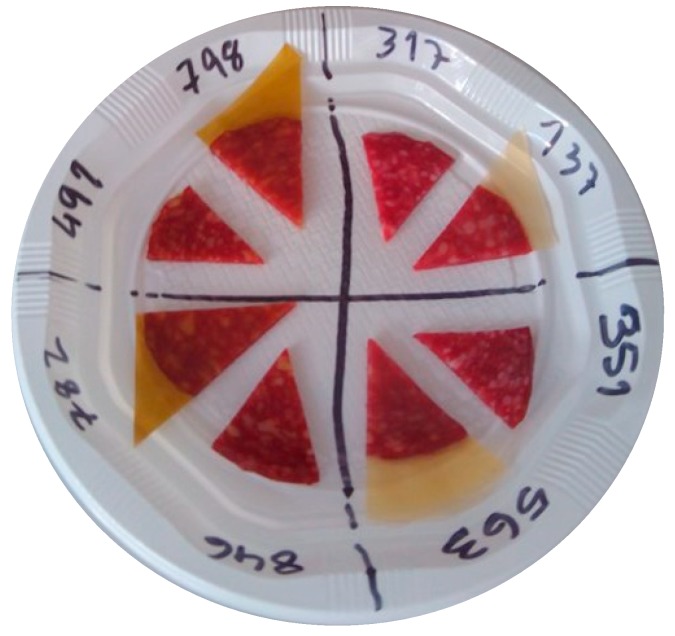
Presentation of the samples to the sensory panel. The code is decrypted in Table 2.

On the second and third sessions, spaced for one week, the salami slices were evaluated after being in contact with the control and the active film for 30 and 90 days. The panel tasted the samples with and without the whey protein film, as represented in Figure 2. As can be observed in Figure 2, random codes were given to the samples to avoid influencing the panel. Table 2 shows the correspondence of the code.

### 2.5. Statistical Analysis

Sensorial data was analysed by mixed model, with tasters as fixed block effect and panel date as random block effect. Tukey’s test was used for Multiple means comparison. For variables only measured at the second panel, an analysis of variance (ANOVA) with tasters as block and samples as fixed effects was used. Tukey’s test was used for Multiple means comparison. 

## 3. Results and Discussion

### 3.1. State of Lipid Oxidation of the Salami Slices Packaged with the Active Film

#### 3.1.1. Hexanal Monitorization

**Figure 3:**
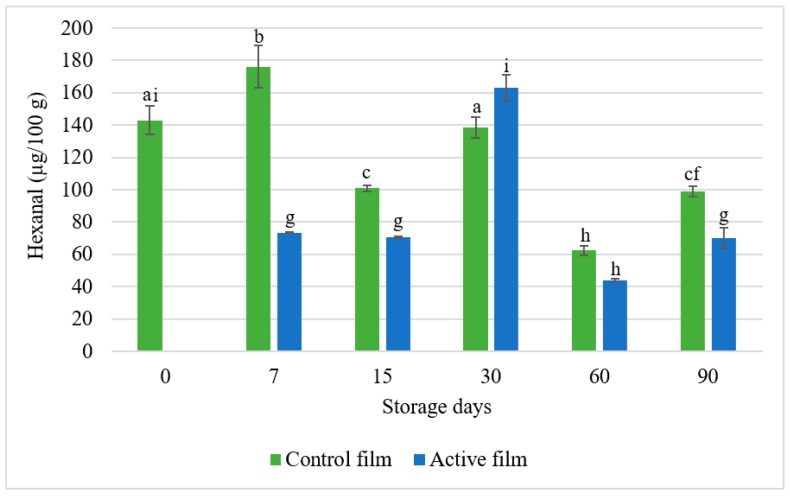
Hexanal assay results between control and active whey protein films stored during 90 days. Control film—whey protein film without the rosemary extract; Active film—whey protein film with 1% (*w*/*w*) rosemary extract. Different letters represent significant differences (*p* < 0.05).

Hexanal (CH_3_(CH_2_)_4_CHO) is the dominant aldehyde formed during lipid oxidation. It is related with the deterioration of organoleptic properties (it is responsible for the rancid aroma and taste of fats) and it is present since the early beginning of the lipid oxidation phenomenon and increases over time, therefore it is considered a good indicator of the lipid oxidation status of some food samples [30,32,33]. With the exception of the 30-day storage time, the results show a reduction in the amount of hexanal in the packaged salami (with both control and active films), when compared with the initial amount of hexanal, in the fresh salami (Figure 3). The higher value on the 30th day of storage presented by the salami packaged with the active film is still lower than the value present in the salami packaged for 7 days. This may indicate that the oxidative status of the salami reached an oxidation peak on the control samples between the 7th day and the 15th day of storage. When compared with the control film, the salami packaged with the active film showed, in general, a superior reduction of the amount of hexanal present through the 90-days period. This proves the effectiveness of the active film on the control of salami lipid oxidation. 

Similar results were obtained by Vilarinho et al. with a polylactic acid (PLA) film incorporated with montmorillonite (MMT) clay Cloisite^®^ Na^+^ nanometric fillers. In this study, the authors found that the active PLA film prevented the formation of hexanal when compared to the PLA control film [34].

Madsen et al. [35] evaluated oil-in-water emulsions, homogenized with rosemary and a rosemary methanolic extract. The emulsions were stored under light exposure conditions for 24 weeks at 19 °C. According to the results of the hexanal assay carried out by the authors, the hexanal content in the emulsion incorporated with the rosemary extract only started to increase from the 6th week, presenting lower values than the control sample, until the 8th week. Concerning the emulsion incorporated with dry rosemary leaves, it presented values lower than 150 mg/kg of hexanal at day 14, contrasting with the 600 mg/kg of hexanal in the control sample and the 750 mg/kg in the sample with the rosemary extract. These authors concluded that although methanol is an efficient extraction solvent of bioactive compounds, it is an antioxidant less powerful than the plant itself, which indicates that the compounds responsible for the antioxidant activity of rosemary were not extracted by the selected solvent [35]. The results of this study indicate that rosemary has in its composition compounds capable of inhibiting or retarding the lipid oxidation of foods with a high fat content, though the chosen solvent is not the most suitable to obtain the antioxidant compounds. In the present study, ethanol was selected as extraction solvent, since it is authorized for the extraction of rosemary according to Directive 2010/67/EU and has a lower toxicity than methanol.

In another study, carried out by Erdmann et al. [36], sausages incorporated with an oil-in-water emulsion of ω-3 and a commercial rosemary extract, were cooked, sliced and stored under a modificated atmosphere (in order to accelerate the oxidation processes) for a 35-day period at 7 °C. When compared to the control sausages, the modified sausages presented less 90% of hexanal, which proves the antioxidant capacity of the rosemary extract [36]. These results are in accordance with the results of this study which, as can be seen in Figure 3, the hexanal content in the salami slices packaged with the active film, is lower than the hexanal content of the salami slices packaged with the control film.

#### 3.1.2. TBARS Assay

**Figure 4:**
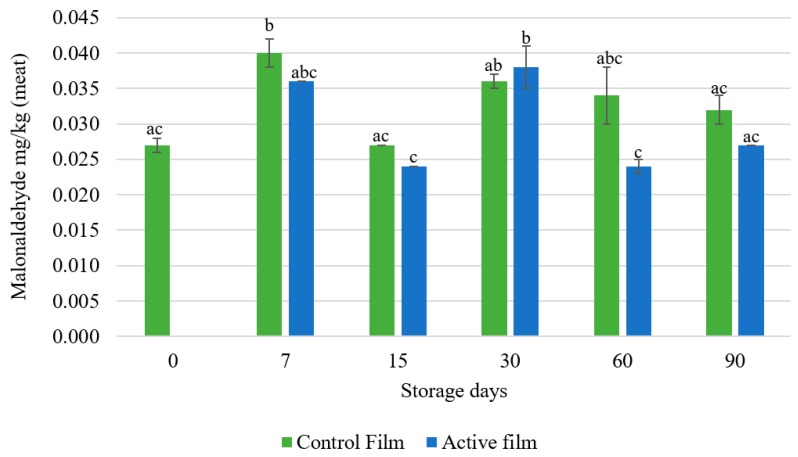
TBARS assay results between control and active whey protein films stored during 90 days. Control film—whey protein film without the rosemary extract; Active film—whey protein film with 1% (*w*/*w*) rosemary extract. Different letters represent significant differences (*p* < 0.05).

The TBARS assay measures the malondialdehyde (CH_2_(CHO)_2_) (MDA) content formed in the reaction between the MDA and thiobarbituric acid ((C_4_H_4_N_2_O_2_S) (TBA). The reaction between the two compounds produces a reddish compound that can be measured in a certain wavelength (500 to 550 nm). It is one of the most used methods to measure the lipid oxidation, especially in meat products [31,32,37]. MDA is an aldehyde formed in the primary oxidation, during the decomposition of unsaturated fat acids to hydroperoxides [38]. As can be observed in the Figure 4, all the salami slices packaged with the active film presented a lower MDA content than the salami slices packaged with the control film, except for the salami packaged for 30 days. The salami slices packaged with the active film for 60 and 90 days show a lower MDA content than the salami slices packaged with the control film, which seems to indicate that the active film can inhibit the salami’s lipid oxidation for long time periods. However, the TBARS assay as some limitations. This assay only measures substances reactive to the thiobarbituric acid which, in the course of lipid oxidation, will continue to be degraded. The increase of the MDA content in the salami slices packaged for 30 days suggest that the oxidative status of the salami slices reached an oxidation peak.

The MDA value increased in the first 7 days of storage. A reduction of the MDA content was observed in the active films at times 15, 60 and 90 days, in which MDA values were relatively stable. Significant differences were only found between the slices packaged with the control film for 7 days Although the MDA values do not decay over time, it is observed that, except for the 30-day time-period, the active film presented lower TBARS value. Also, all the MDA values found, either in the control samples or the active samples, are below 0.5 mg MDA/kg sample, which is the value settle by the consumers in which the off-flavor is precepted [39].

In a study performed by Erdmann et al. [36], a commercial extract of rosemary was emulsified with ω-3 from fish (oil/water emulsion) and it was added to pork sausages. The authors reported that the MDA values in the control sausages began to increase during the first 21 days of storage. In contrast, the sausages homogenized with the rosemary extract presented values 90% lower. Also, a significant increase in the MDA vales was observed in the samples treated with the rosemary extract between the 21st and 35th days. The opposite was observed in the control samples, suggesting that the sausage fat oxidation reached its peak and the oxidation indicators were transformed into other decomposition products [36]. According to the USDA Food Database, when compared to the nutritional values presented by the label of the salami slices (Table 1), the pork sausages have a lower protein content (14.10 g/100 g) and a slightly higher fat content (28.72 g/100 g). Although the nutritional values are not very different, the pork sausages were cooked and packaged under a modified atmosphere (to accelerate the lipid oxidation), whereas, the salami slices were not submitted to any source of heat. This may explain the differences found in the MDA content, which were much higher in the pork sausages.

Also, in the research performed by Hernández-Hernández et al. [40], the efficiency of a rosemary extract against lipid oxidation was demonstrated. The authors tested three rosemary extracts, extracted with two different solvents (chloroform and ethanol) and a mixture of the two extracts. They applied the extracts directly to pork chops, storing the samples at 4 °C for 72 h, protected from light. The lowest MDA concentrations were obtained by the ethanolic extract (0.566 mg MDA/kg) and the samples with chloroform extract showed 0.722 mg MDA/kg, while the control samples had a higher concentration of MDA (1.086 mg MDA/kg) [40].

### 3.2. Sensorial Analyses 

**Figure 5:**
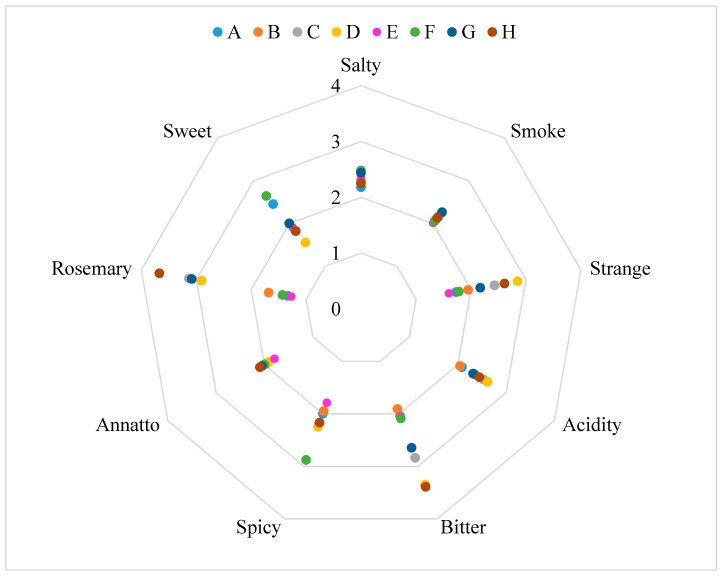
Results of the sensory analysis for taste descriptors. A: Salami slice packaged with the control film during a 90-days storage period evaluated without the packaged; B: Salami slice packaged with the control film during a 90-days storage period evaluated with the packaged; C: Salami slice packaged with the active film during a 90-days storage period evaluated without the packaged; D: Salami slice packaged with the active film during a 90-days storage period evaluated with the packaged; E: Salami slice packaged with the control film during a 30-days storage period evaluated without the packaged; F: Salami slice packaged with the control film during a 30-days storage period evaluated with the packaged; G: Salami slice packaged with the active film during a 30-days storage period evaluated without the packaged; H: Salami slice packaged with the active film during a 30-days storage period evaluated with the packaged.

**Figure 6:**
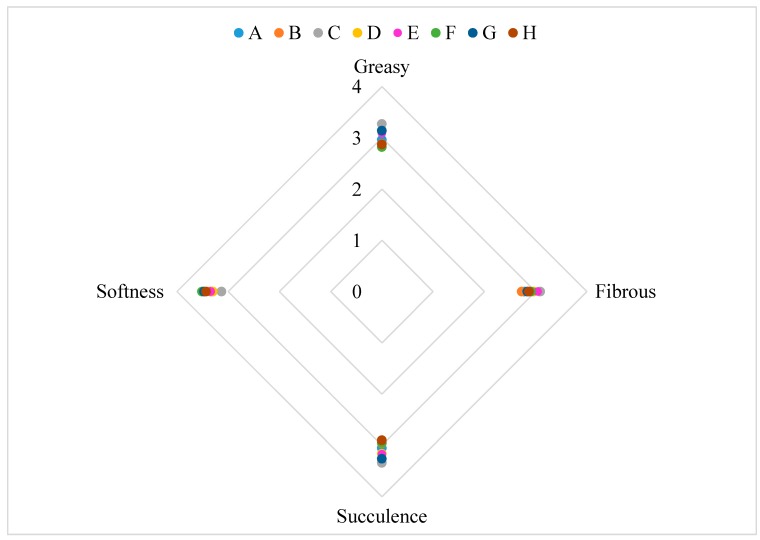
Results of the sensory analysis for texture descriptors. A: Salami slice packaged with the control film during a 90-days storage period evaluated without the packaged; B: Salami slice packaged with the control film during a 90-days storage period evaluated with the packaged; C: Salami slice packaged with the active film during a 90-days storage period evaluated without the packaged; D: Salami slice packaged with the active film during a 90-days storage period evaluated with the packaged; E: Salami slice packaged with the control film during a 30-days storage period evaluated without the packaged; F: Salami slice packaged with the control film during a 30-days storage period evaluated with the packaged; G: Salami slice packaged with the active film during a 30-days storage period evaluated without the packaged; H: Salami slice packaged with the active film during a 30-days storage period evaluated with the packaged.

**Figure 7:**
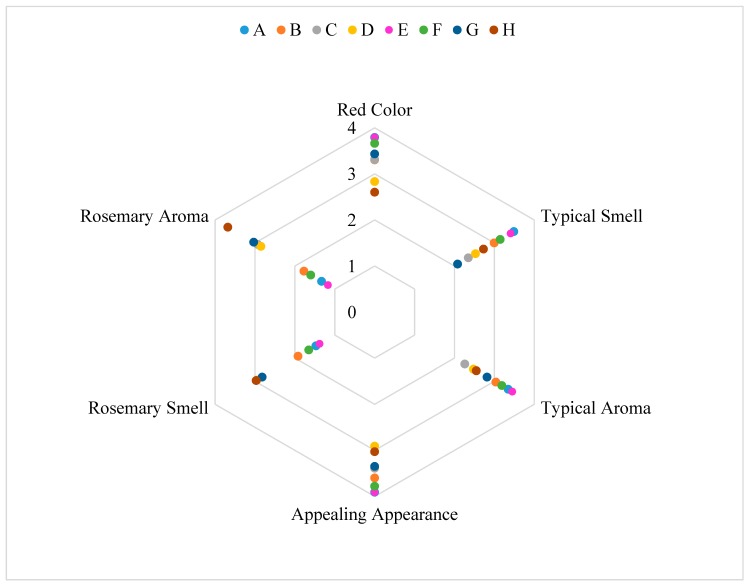
Results of the sensory analysis for other descriptors. A: Salami slice packaged with the control film during a 90-days storage period evaluated without the packaged; B: Salami slice packaged with the control film during a 90-days storage period evaluated with the packaged; C: Salami slice packaged with the active film during a 90-days storage period evaluated without the packaged; D: Salami slice packaged with the active film during a 90-days storage period evaluated with the packaged; E: Salami slice packaged with the control film during a 30-days storage period evaluated without the packaged; F: Salami slice packaged with the control film during a 30-days storage period evaluated with the packaged; G: Salami slice packaged with the active film during a 30-days storage period evaluated without the packaged; H: Salami slice packaged with the active film during a 30-days storage period evaluated with the packaged.

There are significant differences between the samples (Table 3). The differences are between samples tasted with the edible package and the samples tasted without the edible package. The only descriptor that showed significant differences was the “bitter taste”. The results are shown in the form of graphic web in Figure 5, Figure 6 and Figure 7.

Regarding the “bitter taste”, the significant differences are explained by the strong bitter taste present in the rosemary extract. As can be observed in Table 3, the bitter taste increases in the samples C, D, G and H, which are the samples packaged with the active film. The significant differences between the samples can be explained by the migration of active compounds of the rosemary extract incorporated into the active film to the salami samples. The rosemary extract gives foods the strong characteristic bitter taste of the plant. The same can be observed in the other descriptors that showed significant differences, since the samples that have less red color and typical smell are the samples that have been in contact with the active film. The differences are explained by the strong smell and taste of the rosemary extract, directly in contact with the samples. The fact that the samples were tasted with and without the package, does not appear to have any significant difference in taste, texture and other characteristics. The sample considered most appealing was sample A, packaged with the control film, as opposed to the sample H, packaged with the active film tasted with the film for the 30 days time period.

In the sensorial analysis conducted by the authors Frutos & Hernández-Herrero [41], a dressing of sunflower oil, garlic and parsley mixed with a commercial rosemary extract was evaluated. The sensorial panel only detected the presence of the extract in the sample with the highest extract content (6 g/L). In another study conducted by Chammem et al. [42], the addition of a rosemary extract to frying oil, improved the crispness and flavor of potato chips. These authors also noted a pleasant flavor during the frying process in the oil with the rosemary extract. Both Frutos & Hernández-Herrero [41] and Chammen et al. [42], used a higher quantity of rosemary extract in relation to the quantity used in this study. Also, these authors applied the extract directly in the food, while the rosemary extract was only incorporated in the film, with the objective of gradually migrate to the food’s surface. The larger quantity and the direct applicability of the rosemary extract will cause a higher rosemary taste and aroma.

The sensory panel, considered the salami packaged with the active film a little strange, bitter and with a rosemary taste. Nevertheless, all samples were considered to have an appealing appearance (>2.5). It is considered that the overall appreciation of the salami slices packaged with the active film was positive and that the new active packaging could be attractive to the consumer.

## 4. Conclusions

The initially proposed objectives in this study were successfully achieved. The salami slices packaged with the active film showed lower levels of MDA and hexanal, when compared with the control film, except for the salami analysed at the 30th day of storage. Nevertheless, the MDA is below 0.5 mg MDA/kg, the value determinate for the off-flavor perception by consumers. The two lipid oxidation assays seem to indicate that the active film tend to be more protective of the fatty foods against lipid oxidation phenomenon during 60 and 90 days of storage time than the control film (without the rosemary extract). Regarding the sensorial analysis, the panel considered that the packaged samples presented an appealing aspect and that the film preserved the organoleptic characteristics of the salami, adding a slight bitter taste due to the rosemary extract. A more complete sensory analysis must be performed, with a trained panel and a larger number of tasters in order to access the future acceptability of this edible film in the market. Further studies are needed to test the effectiveness against lipid oxidation during longer storage periods. Also, new food matrixes should be applied to test the powerful antioxidant capacity of the rosemary extract. The incorporation of higher percentages of the rosemary extract should be also reviewed and tested in order to increase the antioxidant and antimicrobial capacity of the active film.

## Figures and Tables

**Table 1:** Nutritional composition of salami according to the label information and according to the USDA Nutritional Database [28].

Nutrient	Unit	Value per 100 g		
		Label	USDA	PT
**Water**	g		34.6	38.0
Energy	kcal	352.7	425	422
Protein	g	23.5	21.7	19.5
Total lipid (fat)	g	26.5	37.0	37.6
Carbohydrate	g	4.9	1.20	1.30
Fiber	g		0.00	
Sugars	g		1.20	
**Minerals**				
Calcium (Ca)	mg	-	10.0	25.0
Iron (Fe)	mg	-	1.52	2.30
Magnesium (Mg)	mg	-	22.0	22.0
Phosphorus (P)	mg	-	229	200
Potassium (K)	mg	-	340	140
Sodium (Na)	mg	-	1890	2300
Zinc (Zn)	mg	-	4.20	4.30
**Vitamins**				
Thiamin	mg	-	0.93	0.36
Riboflavin	mg	-	0.33	0.22
Niacin	mg	-	5.60	3.00
Vitamin B-6	mg	-	0.55	0.25
Folate, DFE	µg		2.00	3.00
Vitamin B-12	µg		2.80	1.00
**Lipids**				
Fatty acids, total saturated	g		13.1	12.9
Fatty acids, total monounsaturated	g		18.2	14.9
Fatty acids, total polyunsaturated	g		3.6	4.3
Cholesterol	mg		80.0	80.0

**Table 2:** Decoding of the samples in study presented in Figure 2.

Code	Sample Description
317	Salami slice packaged for 30 days with control film—evaluation without packaging
137	Salami slice packaged for 30 days with control film—evaluation with packaging
491	Salami slice packaged for 30 days with active film—evaluation without packaging
798	Salami slice packaged for 30 days with active film—evaluation with packaging
351	Salami slice packaged for 90 days with control film—evaluation without packaging
563	Salami slice packaged for 90 days with control film—evaluation with packaging
846	Salami slice packaged for 90 days with active film—evaluation without packaging
782	Salami slice packaged for 90 days with active film—evaluation with packaging

**Table 3:** Results of the first tasting for the descriptors for the analyzed samples.

Sample	Salty	Smoke	Strange	Acidity	Bitter	Spicy	Annatto	Greasy	Fibrous	Succulence	Softness	Redcolor	Typicalsmell	Typicalaroma	Appealingappearance
A	2.17^a^	2.00^a^	1.92^a^	1.92^a^	2.00^acde^	2.08^a^	1.92^a^	2.92^a^	2.83^a^	3.00^a^	3.25^a^	3.67^a^	3.17^a^	3.25^a^	3.92^a^
B	2.17^a^	2.00^a^	2.17^a^	2.00^a^	1.92^ce^	2.08^a^	1.92^a^	2.75^a^	2.83^a^	2.83^a^	3.42^a^	3.42^a^	2.92^a^	3.08^a^	3.75^a^
C	2.50 ^a^	2.17^a^	2.58^a^	2.25^a^	2.58^abcde^	2.33^a^	2.08^a^	3.08^a^	3.00^a^	3.42^a^	3.17^a^	3.25^a^	2.25^a^	2.33^a^	3.50^a^
D	2.58^a^	2.42^a^	3.25^a^	2.42^a^	3.25^bd^	2.42^a^	2.08^a^	3.25^a^	2.92^a^	3.42^a^	3.33^a^	2.83^a^	2.42^a^	2.42^a^	3.08^a^
E	2.08^a^	1.83^a^	1.75^a^	2.00^a^	1.92^e^	1.67^a^	1.67^a^	2.92^a^	3.08^a^	3.17^a^	3.42^a^	3.67^a^	3.00^a^	3.08^a^	4.00^a^
F	2.33^a^	2.00^a^	1.75^a^	2.17^a^	2.00^acde^	1.75^a^	1.92^a^	2.92^a^	3.08^a^	3.17^a^	3.75^a^	3.58^a^	2.75^a^	2.83^a^	3.83^a^
G	2.42^a^	2.25^a^	2.17^a^	2.00^a^	2.67^abcde^	2.17^a^	2.00^a^	3.00^a^	2.58^a^	3.25^a^	3.58^a^	3.50^a^	3.33^a^	3.00^a^	3.33^a^
H	2.50^a^	2.25^a^	2.58^a^	2.17^a^	3.42^b^	2.33^a^	2.08^a^	2.92^a^	2.83^a^	3.25^a^	3.50^a^	2.83^a^	3.00^a^	2.83^a^	3.25^a^
*p*	0.51	0.80	0.06	0.93	<0.05	0.52	0.98	0.96	0.93	0.65	0.99	0.29	0.30	0.11	0.40

A: Salami slice packaged with the control film during a 90-days storage period evaluated without the packaged; B: Salami slice packaged with the control film during a 90-days storage period evaluated with the packaged; C: Salami slice packaged with the active film during a 90-days storage period evaluated without the packaged; D: Salami slice packaged with the active film during a 90-days storage period evaluated with the packaged; E: Salami slice packaged with the control film during a 30-days storage period evaluated without the packaged; F: Salami slice packaged with the control film during a 30-days storage period evaluated with the packaged; G: Salami slice packaged with the active film during a 30-days storage period evaluated without the packaged; H: Salami slice packaged with the active film during a 30-days storage period evaluated with the packaged; *p*—*p* value; Different letters indicate significant differences (*p* < 0.05) among salami samples.
